# Comparison Between Simple Batch and Fed-Batch Bioreactor Cultivation of Recombinant BCG

**DOI:** 10.3390/pharmaceutics16111433

**Published:** 2024-11-11

**Authors:** Sarah Mendes, Maria C. P. Gonçalves, Vitoria A. P. Aiex, Ryhára D. Batista, Patrícia Zorzete, Luciana C. C. Leite, Viviane M. Gonçalves

**Affiliations:** 1 Instituto Butantan, São Paulo 05503-900, Brazil; sarah.mendes@usp.br (S.M.); mariacarolinapgoncalves@gmail.com (M.C.P.G.); vitoria.aiex.esib@esib.butantan.gov.br (V.A.P.A.); patricia.zorzete@butantan.gov.br (P.Z.); luciana.leite@butantan.gov.br (L.C.C.L.); 2 Interunits Graduate Program in Biotechnology (PPIB), University of São Paulo, São Paulo 05508-220, Brazil

**Keywords:** *Mycobacterium bovis* BCG, pertussis toxin, onco-rBCG, L-glutamic acid, submerged culture, vaccine

## Abstract

**Background/Objectives**: Tuberculosis continues to be a significant global health concern, causing 1.3 million deaths in 2022, particularly affecting children under 5 years old. The Bacillus Calmette-Guérin (BCG) vaccine, developed in 1921, remains the primary defense against tuberculosis but requires modernized production methods. The recombinant BCG-pertussis strain shows potential in providing dual protection against tuberculosis and whooping cough, especially for vulnerable newborns, and enhanced efficacy against bladder cancer. Implementing submerged cultivation techniques for rBCG-pertussis production can offer increased productivity and standardization. **Methods**: This study explores a fed-batch cultivation strategy with pH-stat control to feed L-glutamic acid through the acid pump into 1 L bioreactor. Three pH values were evaluated for fed-batch and a simple batch without pH control was done for comparison. The viable cell concentration was compared before and after freeze-drying samples harvested during the cultures. **Results**: L-glutamic acid was identified as the preferred substrate for rBCG-pertussis. While the fed-batch strategy did not enhance the maximum specific growth rate compared to simple batch cultivation, it did improve the specific growth rate after day 4 in the pH 7.4-controlled fed-batch cultures, thereby reducing the cultivation time. Fed-batch cultures controlled at three pH levels exhibited lower optical density than the simple batch, although the viable cell counts were similar. Notably, samples harvested after day 8 from the simple batch cultures showed a reduction in CFU/mL after freeze-drying, whereas all fed-batch samples exhibited high recovery of viable cell counts post lyophilization. **Conclusions**: The additional glutamate supplied to the fed-batch cultures may have protected the cells during the lyophilization process.

## 1. Introduction

Tuberculosis is one of the main infectious diseases worldwide, and it was the second leading cause of death for infectious diseases in 2022, only behind COVID-19, causing around 1.3 million deaths [1]. One in five children does not survive tuberculosis development [2], and children under 5 years old are the main risk group [3]. *Mycobacterium tuberculosis* is the bacterium causing tuberculosis, transmitted through respiratory droplets from infected hosts [3].

In 1921, Calmette and Guérin attenuated *Mycobacterium bovis* through successive passages in culture medium and generated the Bacillus Calmette-Guerin (BCG) vaccine against human tuberculosis, since *M. bovis* and *M. tuberculosis* species have 90% homology [4]. Currently, BCG immunization is one of the most adopted vaccination strategies worldwide and the sole vaccine available against tuberculosis [5]. Since 1976, BCG has also been used as an immunotherapeutic product (onco-BCG) to treat non-muscle invasive bladder cancer using a 10-times higher dose than the vaccine, and it remains the standard treatment for it nowadays, with a reduction of disease progression and minimizing its recurrence [6,7].

More recently, recombinant BCG (rBCG) strains have been investigated as antigen-presenting systems for vaccination against other infectious diseases because of BCG’s ability to stimulate an innate immune response and deliver antigens directly to macrophages and dendritic cells [8]. Our laboratory has developed the rBCG-pertussis strain using *M. bovis* BCG Moreau bacilli, which expresses a nontoxic mutant gene of the S1 subunit of *Bordetella pertussis* toxin [9,10].

*Bordetella pertussis* is a bacterium that infects the respiratory tract, potentially causing whooping cough in non-immunized individuals. The most affected group is non-immunized newborns [11]. Currently, immunization against *B. pertussis* is performed with the DTP vaccine, which prevents diphtheria, tetanus, and whooping cough and is administered at 2, 4, and 6 months of age. Pertussis toxin is the most important antigen of *B. pertussis*, composed of five subunits: S1 contains its active site and shows an immunogenic response in animals, and subunits S2 to S5 organize to form the host cell receptor binding site [12,13]. rBCG-pertussis protected neonatal mice against both tuberculosis and pertussis [9,10]. In addition, rBCG-pertussis showed better results against bladder cancer in terms of tumor weight reduction and survival time than the conventional BCG in mouse model [14]. Also, an in vitro model using human blood cells challenged with the rBCG-pertussis strain has shown improved immune activation profiles when compared to the conventional BCG strain [15].

The current method for producing BCG is based on static cultivation in flasks, where the bacilli form a pellicle on the surface of the medium [16]. The static cultivation in flasks is a labor-intensive and not easily standardized process, being an important cause for the shortage of the BCG vaccine [16] and onco-BCG across the world [17]. BCG submerged cultivation in bioreactors would allow greater productivity and standardization. Nonetheless, the first systematic comparison between BCG produced as pellicle or in disperse shaken flasks was only recently published [16], and there are very few reports on BCG submerged cultivation in bioreactors [18,19,20,21,22,23]. Van Hemert was the first to report that homogeneous cultivation in a bioreactor was possible in the presence of detergent [18]. This work employed either Triton WR 1339 or Tween-80 in Ungar medium [24]. Kim used a modification of Proskauer and Beck medium [25] containing glucose and Tween-80 [19]. Three of the works employed Sauton medium [26], either without detergent [20,22] or with Tween-80 to avoid bacilli aggregation and antifoam C emulsion to prevent foam formation [21]. More recently, *M. bovis* BCG Danish was cultivated in 1-L bioreactors with different culture media containing 0.5% glycerol and 0.2% Tween-80, and it was shown that Roisin’s minimal medium and Middlebrook 7H9 with 10% OADC showed similar results [23].

BCG vaccines produced in bioreactors were reported to pass all tests of safety and virulence [19]. Also, they exhibited similar efficacy against mouse challenges with *M. tuberculosis* when compared to the classical surface-grown vaccine [21]. In addition, the tuberculin produced from the supernatant of BCG bioreactor cultures presented no differences to the product obtained in static flasks [20]. Most importantly, the comparison between BCG produced as pellicle or in disperse shaken flasks showed no differences, indicating that the bioreactor manufacturing process should be considered as a replacement for the traditional pellicle growth method [16]. However, the low price of the vaccine and regulatory issues, among other factors, can be barriers to invest in process modernization [27]. On the other hand, the bioreactor manufacturing process has already been implemented in the production of new TB vaccine candidates [16]. Similarly, a new and improved product as rBCG-pertussis could stimulate the investments necessary to modernize the production process of both vaccine and immunotherapeutic products, bringing positive impacts to public health, as the new rBCG-pertussis vaccine would protect the age group under 6 months, which is the most vulnerable to *B. pertussis* infection, and a new onco-rBCG would contribute to solving the shortage problems related to the onco-BCG supply chain [17].

Therefore, the study of feeding strategies in submerged cultures of rBCG-pertussis with benchtop bioreactors can lead to an increase in cell concentration and in the yield of the process. In addition, the application of the pH-stat control method can provide greater knowledge about the behavior of rBCG-pertussis in different pH ranges.

The aim of this work is to develop a fed-batch cultivation with the pH-stat control method using L-glutamic acid, which was shown here to be the preferential substrate of BCG using the Monod model of cell growth.

## 2. Materials and Methods

### 2.1. Inoculation and Cultivation of rBCG-Pertussis

The rBCG-pertussis strain, a recombinant strain of the original BCG Moreau and lysine auxotrophic, was used [10]. The strain was inoculated into a 250 mL Erlenmeyer flask containing 50 mL of 7H9 modified medium [28] incubated in a shaker at 37 °C and 125 rpm. After 96 h of cultivation, with the optical density (OD) close to 4, the calculation was made to determine the necessary inoculum volume for the bioreactor cultivation to start with the OD around 0.2, which was measured using a Hitachi U-5100 spectrophotometer at 600 nm. Culture samples were diluted in phosphate-buffered saline with 0.05% Tween-20 (PBS-T) when the OD was higher than 0.6.

The cultivations were performed in a 1-L bioreactor (Ralf Plus Duet, Bioengineering, Wald, Switzerland), equipped with three Rushton-type impellers and a working volume of 500 mL. Prior to inoculation, the bioreactor was sterilized with phosphate-buffered saline (PBS) at 121 °C for 30 min. Before cultivation, the PBS was aseptically drained, and the 7H9 modified medium, previously sterilized by filtration at 0.22 µm, was aseptically transferred into the bioreactor.

The 7H9 modified medium [28] was composed of Middlebrook 7H9 broth (BD Difco, Sparks, MD, USA) supplemented with glycerol (20 g/L), glucose (2 g/L), ammonium sulfate (0.5 g/L), magnesium sulfate heptahydrate (0.25 g/L), zinc sulfate heptahydrate (4 mg/L), and L-glutamic acid (2.5 g/L). Tyloxapol (0.5 g/L) was used as a surfactant to prevent cell aggregation, and the initial pH was adjusted to 6.7. To avoid excessive foam formation, superficial aeration was performed.

Control cultures without feeding (simple batch) were performed, and three pH values were evaluated in pH-stat mode by feeding around 350 mL of 7.5 g/L L-glutamic acid solution complemented with tyloxapol (0.5 g/L) through the acid pump to control the pH at the desired value. Two replicates were performed for each culture condition, and all cultures were performed at 37 °C and 20% dissolved oxygen.

### 2.2. Viable Cell Counting

Daily samples were collected to determine the concentration of viable cells by plating and counting colony-forming units per mL (CFU/mL). The samples were serially diluted in PBS-T (1:10^3^, 1:10^4^, 1:10^5^, and 1:10^6^) and plated on Middlebrook 7H10 OADC (BD Difco, Sparks, MD, USA) agar plates.

### 2.3. Quantification of Glucose, Glycerol, and Glutamate

The daily samples were centrifuged at 4500× *g* (Labofuge 400R, Heraeus, Germany) for 15 min, and the supernatant was analyzed for the concentration of glucose and glycerol using High-Performance Liquid Chromatography (HPLC). The HPLC equipment used was Agilent 1260 Infinity with the Aminex HPX-87H column (Bio-Rad, Hercules, CA, USA). The mobile phase was 5 mM H_2_SO_4_ at 0.6 mL/min and 60 °C. Glucose and glycerol were detected by a refraction index detector and organic acids by a UV detector at 210 nm.

In addition, the daily supernatant samples were quantified for glutamate using a biochemical analyzer YSI 2700 Series (YSI Life Sciences, Yellow Springs, OH, USA) following the manufacturer’s instructions.

### 2.4. Specific Growth Rate and Monod Model of Growth Kinetics

For all cultivation procedures, the maximum specific growth rate (μ_max_) was calculated by the angular coefficient of linear regression fit of Ln(OD/OD_initial_) versus time from day 0 to 4 and µ in the deceleration phase (µ_dec_) after day 4 to the beginning of the stationary phase.

The Monod model was adjusted to all three main substrates: L-glutamic acid, glucose, and glycerol for rBCG-pertussis growth using simple batch data, according to Equation (1):

(1) $$\mu =\frac{\mu_{max}\cdot S}{K_{s}+S}$$

where µ is the specific growth rate (day^−1^), µ_max_ is the maximum specific growth rate (day^−1^), S is the substrate concentration (g/L), and K_s_ is the substrate concentration that results in half of the µ_max_ value. The solver tool from Excel was employed to estimate the K_s_ and µ_max_ values for every substrate minimizing errors between the observed values and calculated values for µ and K_s_.

### 2.5. Freeze-Drying

From the fifth day until the end of the bioreactor cultivations, samples were collected for freeze-drying. The samples (1 mL) were centrifuged at 4500× *g* (Labofuge 400R, Heraeus, Germany) for 15 min at 4 °C, the supernatant was discarded, and the cell pellet was resuspended in 1 mL of lyophilization solution, which contained mannitol (40 g/L), saccharose (2.5 g/L), sodium glutamate (7.5 g/L), trehalose (5 g/L) and tyloxapol (0.5 g/L) [29]. The samples were charged in the freeze-dryer (FreeZone^®^ Triad^TM^ Freeze Dry System, Labconco, Kansas City, MO, USA) and were freeze-dried following the method described by Jin et al., 2011 [29].

## 3. Results

### 3.1. Simple Batch and Preferential Substrate of rBCG-Pertussis

**Figure 1:**
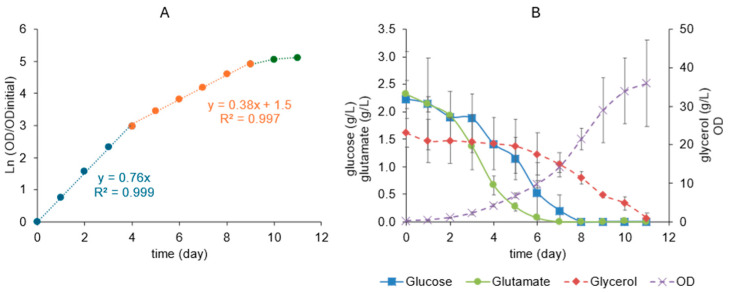
(**A**) Cell growth rate during simple batch cultivation of rBCG-pertussis in a bioreactor with 500 mL of 7H9 modified medium. Average curves (n = 2) are presented, and specific growth rates (µ) are given by angular coefficients of linear regression fit of Ln(OD/OD_initial_) versus time: μ_max_ in blue and µ_dec_ in orange. The stationary phase points are in green. (**B**) Concentration of glucose, glutamate, and glycerol and OD (mean and deviation, n = 2).

A simple batch bioreactor cultivation of rBCG-pertussis was performed to be considered the control group. During the first four days of cultivation, the maximum specific growth rate (µ_max_), 0.76 day^−1^, was reached. After this period, the specific growth rate reduced to 0.38 day^−1^ up to day 9, when the stationary phase started (Figure 1A).

The concentration of the main substrate sources was analyzed daily during the cultivation. The glutamate concentration levels decreased to less than 0.5 g/L on day 6. Glucose was around 1.25 g/L at that same day, while glycerol was still very close to its initial concentration, around 18 g/L (Figure 1B). The cell growth during the simple batch culture reached OD 36 ± 11 on day 11, when all carbon sources were exhausted, while OD 22 ± 2 on day 8 and 29 ± 6 on day 9 were reached (Figure 1B).

**Figure 2:**
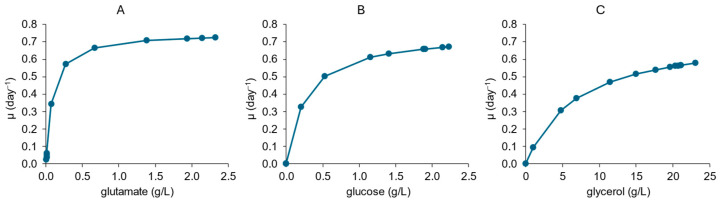
Monod model for rBCG-pertussis growth kinetics considering (**A**) glutamate, (**B**) glucose, and (**C**) glycerol as substrates during simple batch cultivation of rBCG-pertussis in a bioreactor with 500 mL of 7H9 modified medium. Average curves (n = 2) are presented.

The Monod model was adjusted for each substrate: glutamate, glucose, and glycerol (Figure 2), and the K_s_ values were estimated. The results showed that the K_s_ value for glutamate was the lowest (0.085 g/L), followed by glucose (0.26 g/L) and glycerol (6.9 g/L), which points to a glutamate relevance for rBCG-pertussis metabolism. Also, the µ_max_ estimated by the Monod model fitting was 0.72 day^−1^, similar to that calculated by the angular coefficient of the linear regression fit of the curve Ln(OD/OD_initial_) versus time, 0.76 day^−1^ (Figure 1A). For glucose and glycerol, the µ_max_ estimated by the Monod model fitting were 0.67 day^−1^ and 0.58 day^−1^, respectively. These results indicate that it is possible to assume that glutamate is the preferential substrate for rBCG-pertussis growth, and it is limiting the cell growth in the cultivation conditions evaluated here.

### 3.2. Fed-Batch Cultivation Using pH-Stat Strategy

Based on the results of the simple batch cultivation, we hypothesized that the feeding of L-glutamic acid during rBCG-pertussis cultivation could be a way to increase the duration of the exponential growth phase and, consequently, increase the cell growth and viable cell count. The fed-batch cultivations were performed using the pH-stat strategy for a period of the culture, controlling the pH at 7.0, 7.2, and 7.4 until the feeding solution ended. After that, the batch followed its natural pH variances until the stationary phase was reached.

**Figure 3:**
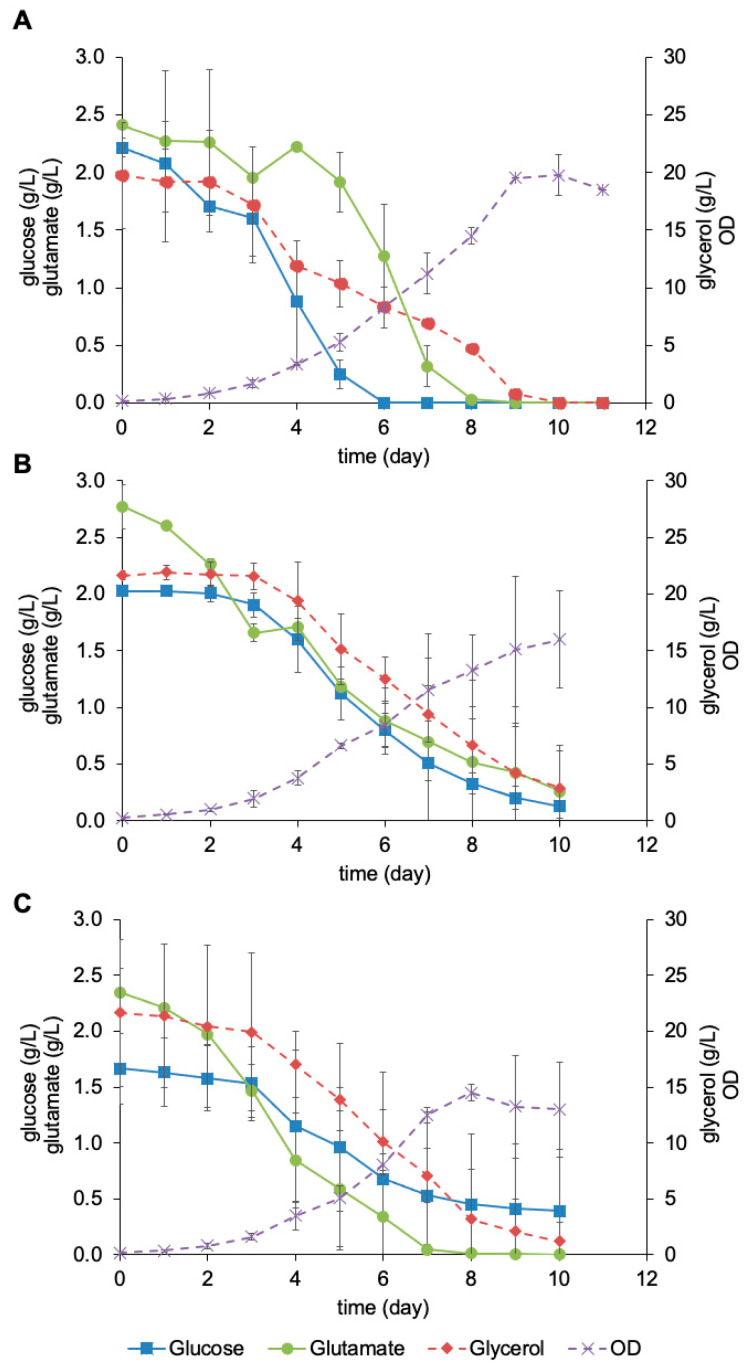
Concentrations of glucose, glutamate, and glycerol and cell growth in a fed-batch bioreactor cultivation of rBCG-pertussis using the pH-stat strategy at pH 7.0 (**A**), pH 7.2 (**B**), and pH 7.4 (**C**). Mean (n = 2) and standard deviation values are shown.

The profile of substrate consumption in the fed-batch cultures can be seen in Figure 3. For the fed-batch controlled at pH 7.0, it was possible to notice an accumulation of glutamate between days 3 and 5, while glucose and glycerol were exhausted, respectively, on days 6 and 10 (Figure 3A). A slight accumulation of glutamate was also observed between days 3 and 4 on the fed-batch controlled at pH 7.2, and none of the substrates were totally consumed in this condition (Figure 3B). For the fed-batch controlled at pH 7.4, glutamate was the only substrate completely consumed on day 8, and no accumulation occurred (Figure 3C).

### 3.3. Comparison Between Simple Batch and Fed-Batch Cultivations

For all cultures, the exponential growth phase lasted through the first four days of the cultivation, so the L-glutamic acid feeding was not able to prolong the duration of this growth phase. The µ_max_ at pH 7.4 was 0.79 day^−1^, only slightly higher than the control, 0.76 day^−1^, while the lowest µ_max_ value was observed in the fed-batch controlled at pH 7.2, 0.73 day^−1^; consequently, the doubling time (Td) was a little higher than the others (Table 1). On the other hand, important differences were observed in the µ_dec_ values and duration of the deceleration growth phase (Table 1). The duration diminished from 5 days in the simple batch and fed-batch at pH 7.0 to 4 and 3 days, respectively, in the fed-batch at pH 7.2 and 7.4. The highest µ_dec_ value was reached in the fed-batch at pH 7.4, which also showed the shortest duration (Table 1).

**Figure 4:**
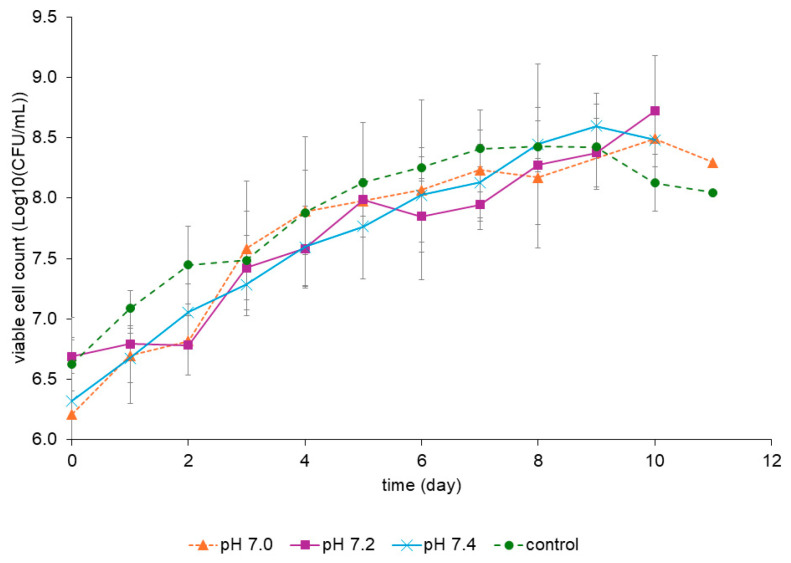
Viable cell counts in fed-batch cultures of rBCG-pertussis using the pH-stat strategy at pH 7.0, pH 7.2, and pH 7.4 compared to the simple batch (control). Mean values (n = 2) and standard deviation are shown.

Although the fed-batch controlled at pH 7.0 reached a maximum OD of 20 ± 1.8, which is superior to the values measured for pH 7.2 and 7.4, it showed the lowest value of viable cell count, 3.1 × 10^8^ CFU/mL (Table 1). The fed-batch controlled at pH 7.2 showed an increase in the maximum viable cell count, reaching 1.1 × 10^9^ CFU/mL, higher than the control group and the fed-batch at pH 7.4, respectively, 3.8 × 10^8^ CFU/mL and 4.6 × 10^8^ CFU/mL. It is noteworthy that the simple batch reached the highest OD value, 36, at the end of the cultivation but not the highest viable cell count. The lack of correlation between OD values and CFU/mL was probably due to cell aggregation. Although clumps containing macroscopic aggregates were not observed in the cultures, because we used superficial aeration, the scan electron microscopy showed there were microscopic aggregates, as well as single cells. These microscopic aggregates would form only one colony on agar plates, but this microscopy technique is a qualitative analysis that cannot be applied to determine the amount of cell aggregates. The analysis of viable cell counts throughout the entire time course of the cultures indicates that all values were in the same range considering the deviation (Figure 4). In addition, the results were similar for all conditions, and, in general, the values were within the deviation (Table 1 and Figure 4).

### 3.4. Influence of the Harvest Moment on the Recovery of Viable Cells After Freeze-Drying

**Figure 5:**
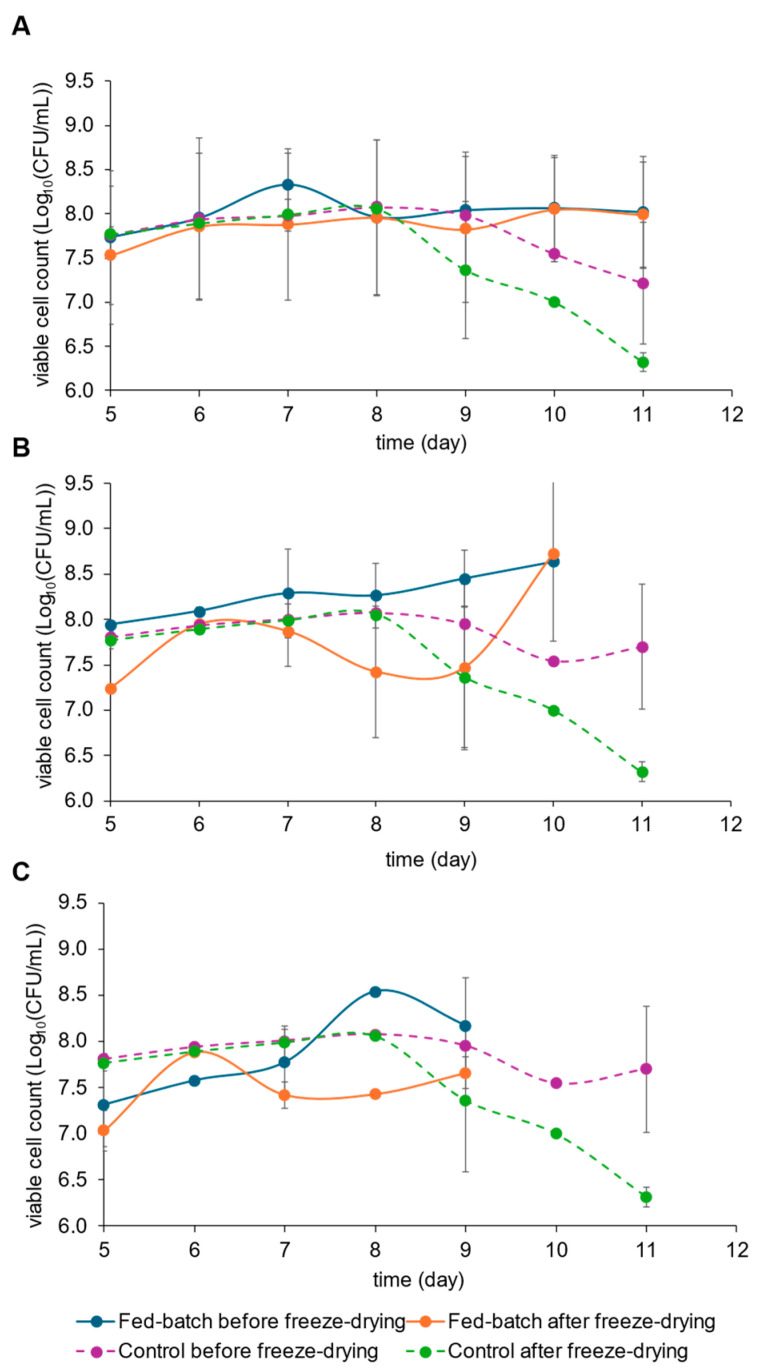
Viable cell counts before and after freeze-drying of fed-batch cultures of rBCG-pertussis in a bioreactor controlled at pH 7.0 (**A**), pH 7.2 (**B**), and pH 7.4 (**C**), all compared to the viable cell count of the simple batch (control). Mean values (n = 2) and standard deviation are shown.

The viable cell count is an important parameter to consider for manufacturing both vaccine and onco-BCG, and the final viability will influence the yield of each production lot. Since the final product needs to be freeze-dried for the final formulation, we investigated if the moment of harvesting would influence the recovery of viable cell counts before and after lyophilization. The results of this analysis are shown in Figure 5.

The viable cell counts before and after freeze-drying remained the same until day 8 of cultivation for the simple batch. After this period, there was a progressive decrease of CFU/mL until the end of the culture (Figure 5). For the fed-batch cultivations, the profile varied according to the pH value. Hence, the viable cell counts before and after freeze-drying were very similar in all days of the fed-batch cultivation at pH 7.0; thus, this strategy was able to maintain the cell viability after freeze-drying of the samples harvested up to the end of the culture (Figure 5A). The viable cell counts after lyophilization stayed lower than the values before lyophilization throughout the fed-batch cultures controlled at pH 7.2 and 7.4 (Figure 5B,C). The opposite of that was observed in the simple batch, as the CFU/mL decrease at the end of the culture was not observed in the fed-batch at pH 7.2 (Figure 5B), and it was not clear if it would happen at pH 7.4 (Figure 5C). However, it should be noted that these two fed-batch cultures were shorter than the simple batch, and if they had lasted longer, the results of the samples taken after days 9–10 could have been different.

## 4. Discussion

The cell growth of rBCG-pertussis, as observed in the control culture without feeding, was accompanied by an increase in pH due to glutamate consumption. This increase persisted until glutamate depletion on day four, reaching pH values close to 7.6; after which, it began to decline due to glucose and glycerol consumption. Therefore, feeding L-glutamic acid through the acid pump to maintain the culture pH could be a strategy to supply this substrate according to the microorganism demand and to improve cell growth. Additionally, this could be beneficial to extend the duration of the exponential growth phase, which was observed during days 0 to 4, the same period in which glutamate was completely consumed in the simple batch culture.

Although the fed-batch cultures did not show an increased cell growth or an extension of the exponential growth phase, the µ_Max_ values observed in Table 1, ≥0.73 day^−1^, were more than twice those observed in the literature, 0.33 day^−1^ [22]. Furthermore, the small differences among the µ_Max_ values indicated that the L-glutamic acid feeding was not sufficient to reduce the doubling time during the exponential growth phase, which remained within the range of 20 h to 23 h in all conditions evaluated. One possible explanation for this result could be the hydrophobic nature of the cell wall surface of mycobacteria, including BCG, which hinders the transport of nutrients across the bacterial cell membrane [30]. Therefore, even with an increased supply of the BCG preferred substrate, i.e., L-glutamic acid, its consumption remained limited by the hydrophobicity characteristic of BCG cell wall. Another possibility is that other nutrients could be limiting the cell growth, and they should be added in the feeding solution together with L-glutamic acid.

Some solutions could be proposed to deal with the nutrient transport limitation. A high molecular mass fraction (>30 kDa) containing RpfA and RpfB was reported to promote an increase in bacterial growth [31]. Thus, the addition of these Rpf muralytic enzymes could lead to a controlled hydrolysis of the cell wall and facilitate nutrient transport. A genetic manipulation to insert porin MspA from *Mycobacterium smegmatis* into *M. tuberculosis* and *M. bovis* BCG was reported to increase twice the glucose uptake and accelerate the cell growth of *M. bovis* BCG [32]. However, this genetic manipulation of BCG could have other consequences and even alter aspects of the strain attenuation, since it would interfere with the metabolism and improve in vivo the fitness of the microorganism. Another possible solution to increase the growth rate, without necessarily changing the nutrient transport, would be the use of metabolic engineering tools to determine nutrient flows through the pathways and identify combinations of substrates and/or new substrates that stimulate cell growth. Recently, the nitrogen flow distributions for amino acid and nucleotide biosynthesis in mycobacteria were analyzed, and glutamate was shown to be the central node for nitrogen metabolism [33]. This platform could also be used to find nutrients to optimize the growth rate of *M. bovis* BCG.

An important parameter for the final formulation of the BCG vaccine or onco-BCG immunotherapeutic product is the concentration of viable cells, as cells need to be viable to generate an effective immune response [22,34]. As observed in Table 1, the simple batch showed the highest OD value among all cultures conducted, reaching OD 36 ± 11. Reported values in the literature show a maximum OD of 2.2 [21] and 8.0 [23] in bioreactors, while a maximum OD of 4.0 was observed in shaken flasks [16]. Regarding the concentration of viable cells, the results were similar for simple batch and fed-batch, with maximum mean values between 3.1 × 10^8^ and 1.1 × 10^9^ CFU/mL, whereas Kim et al. achieved a maximum concentration of viable cells of 5 × 10^8^ CFU/mL [19] and Dietrich et al. of 1.01 × 10^9^ CFU/mL [21]. Therefore, the cultures of rBCG-pertussis conducted with Middlebrook 7H9 modified culture medium achieved greater cell mass than those reported in the literature with high concentrations of viable cells.

Another important factor to consider is the concentration of viable cells before and after lyophilization of rBCG-pertussis culture samples, as this is a necessary step for the final formulation of the vaccine or immunotherapeutic product. In the simple batch culture, when samples were collected between days 5 and 8 of cultivation, it was possible to maintain the same concentration of viable cells before and after lyophilization. However, after this period, and until the end of the cultivation, there was a decrease in the concentration of viable cells after lyophilization. For cultures fed with L-glutamic acid and controlled at pH 7.0 and 7.2, a complete recovery of the viable cell concentration was observed after lyophilization even when samples were collected on days 11 and 10 of the cultivation, respectively, which could indicate a delayed protection of the cells after lyophilization. In contrast, in cultures fed with L-glutamic acid and controlled at pH 7.4, this behavior was not observed, with a decrease in the viable cell count noted after lyophilization in the samples collected after the seventh day of cultivation. Considering that L-glutamic acid is part of the lyophilization solution [29], this amino acid is important for cell preservation, and feeding it in the early days of cultivation, as done in the cultures controlled at pH 7.0 and 7.2, appeared to provide an intracellular reserve of nutrients that possibly improved the resistance of cells to the freeze-drying process.

This study has some limitations. Despite the literature identifying the important role of glutamate as the central node for nitrogen metabolism [33] and the simple batch data showed it was the limiting substrate, the feeding strategy using this amino acid did not promote higher cell growth, indicating that other nutrients might be involved. Accordingly, the model applied here might not be the ideal one to describe rBCG-pertussis growth kinetics in the presence of three substrates (glutamate, glucose, and glycerol), and multi-substrate kinetic models would better represent the results. The study primarily focused on the viable cell count recovery immediately after freeze-drying, and further assays are needed to assess the long-term stability in various storage conditions. Nonetheless, a previous work showed that the best recovery after lyophilization also resulted in high stability after 4 weeks [29]. Finally, the performance and application of the rBCG-pertussis strain still has to be evaluated.

Overall, the higher viable cell concentrations reached in fed-batch cultures compared to the simple batches were not high enough to offset the increased operational complexity of the fed-batch compared to the simple batch cultivation. Therefore, simple batch cultivation remains more advantageous than the fed-batch cultures performed in the conditions evaluated here. Nonetheless, the precise quantification of viable BCG count has long been recognized as difficult due to cell aggregation [20,23], and the plating method used here could underestimate the CFU/mL values. Further investigations on other fed-batch strategies should be done to demonstrate if it is possible to increase the BCG cell density, as well as the viable cell concentration, and define if the lack of correlation between OD and viable cell count is due to cell aggregation.

## Figures and Tables

**Table 1:** Results for the maximum optical density (OD_max_), maximum colony-forming units per mL (max CFU/mL), doubling time (Td) from 0 to 4 days, µ_max_ for the fed-batch cultures and control batch, which were calculated from 0 to 4 days, and µ_dec_ from day 4 up to the beginning of the stationary phase, as indicated in parentheses.

Culture	OD_max_	Max CFU/mL	Td (h)	µ_max_ (day^−1^)	µ_dec_ (day^−1^)
pH 7.0	20 ± 1.8 (day 10)	3.1 × 10^8^	22 ± 1	0.76 ± 0.03	0.35 (days 4–9)
pH 7.2	16 ± 3.0 (day 10)	1.1 × 10^9^	23 ± 1	0.73 ± 0.03	0.31 (days 4–8)
pH 7.4	15 ± 1.0 (day 8)	4.6 × 10^8^	21 ± 1	0.79 ± 0.03	0.43 (days 4–7)
Control	36 ± 11 (day 11)	3.8 × 10^8^	22 ± 1	0.76 ± 0.02	0.38 (days 4–9)

## Data Availability

The original data presented in the study are openly available in Sapitentia Repositório do Instituto Butantan at https://repositorio.butantan.gov.br/handle/butantan/5403 (accessed on 3 November 2024).
